# Medical Opinion and Movement

**Published:** 1911-07-08

**Authors:** 


### Medical Opinion and Movement.

### Causation of Cyclic Vomiting.

The curious condition present in children known as cyclic or recurrent vomiting is the subject of a paper by Dr. Mellanby in the *Lancet* of July 1. Comparatively common though the condition is, possibly it is not very well recognised in the profession. Only during the last fourteen or fifteen years has it attracted much attention anywhere, and it cannot yet be said to have found an established place in text-books on diseases of children. Yet it is a condition which may at times cause considerable anxiety to the relatives of a child and tax the powers of diagnosis of the medical attendant. The writer of this review has twice known of cases operated upon for supposed intestinal obstruction, one of which ended fatally. The disease left to itself, serious though occasionally the state of the child may appear to be, rarely ends fatally. A surgical operation may be sufficient to overtax the child's powers of resistance to shock, and therefore, it is scarcely necessary to remark, should if possible be avoided. It is not often, however, that cases are likely to arise in which intestinal obstruction is thought to be present. The history of previous attacks will generally be too definite to lead to the supposition that any such condition endangering the patient's life is present.

### Course and Prognosis of Cyclic Vomiting.

Although the danger to life is small, the attacks may be so frequent that the child may almost be said to be a chronic invalid, and even where the attacks are comparatively rare when they do occur they may be so severe as to give rise to continual dread of their recurrence. It is important therefore that the nature of the affection should if possible be ascertained and means adopted to render the sufferer less subject to attacks. The paper of Dr. Mellanby is a valuable contribution to the subject. He attacks the view widely held that an acidosis, involving the excretion in the urine of β *oxybutyric* acid, aceto-acetic acid, and acetone, is the cause. He goes so far as to say that the importance which "the medical mind has assigned" to the presence of these bodies in some pathological conditions "is nothing short of ludicrous." It has always seemed to us that the view taken upon this subject has been extremely narrow. We often speak highly of the scientific mind, but it is to be feared that occasionally a small fact looms very large before the eyes of the scientist and blocks the view to other facts more important behind it. By no means uncommonly effect is, we fear, without good reason made to appear as the cause. However that may be, Dr. Mellanby shows that in a case of recurrent vomiting which was under the care of Dr. Box at St. Thomas's Hospital during the period of health there was no acetone in the urine. "The acetone only appeared in the urine after the attack had been fully established." The child, however, always excreted creatin, and there was a gradual rise in the proportion of creatin to creatinin before the attack of vomiting.

### Acidosis.

Another point of interest was established: the well-known statement is referred to that the presence of acidosis is due to inability of the body to oxidise fatty acids. In order to test the child's power to oxidise fatty acids he was given cod-liver oil and extra quantities of cream and bacon fat. He relished the fat and put on considerable weight. He certainly had the power therefore to assimilate fatty acids. Dr. Mellanby then proceeds to reason as to the importance of creatin. Apparently an abnormal quantity of creatin is present in the urine in various pathological conditions, especially in some diseases of the liver, such as cancer. It is shown that there may be a relation between creatin and acidosis, and that creatin and acetone have been observed to appear together in the urine. Both may be due to some abnormality in the metabolism of the liver-cells, and although acidosis was not the cause of the vomiting, it is suggested that the presence of creatin in the urine means defective physiological working of the liver, due possibly to the presence of some intestinal toxin, the potency of which periodically becomes so marked as to make the child very ill. The well-known view is also mentioned that one of the main functions of the liver is to neutralise intestinal toxins, and that in the absence of glycogen there is a diminution in the poison neutralising effect. It is suggested that to give large quantities of glucose might be of value in conditions in which acidosis is present in order to aid the storing up of glycogen. When the boy, the subject of recurrent vomiting, was passing acetone in the urine the giving of glucose apparently almost immediately led to the disappearance of acetone. Possibly creatin in the urine of a child of six may not be so uncommon as Dr. Mellanby supposes. However that may be, Dr. Mellanby appears to have effectually disposed of the acidosis theory of recurrent vomiting.

### Skin Ulcerations and Massage.

In the *New York Medical Journal*, Dr. E. F. Cyriax explains his method of treating simple inflammatory ulcers of the skin by manipulations. He describes those manipulations in detail. They consist in: I. Local manipulations on and around the ulcers—(1) stationary vibrations around the ulcer; (2) concentrating vibrations; (3) vibrations with simultaneous to-and-fro movement of the skin; (4) nerve frictions; (5) circular kneadings and frictions. II. Local manipulation of the affected limb or part of the body. III. General massage. The immediate effects are—Subjectively, removal or diminution of pain; objectively, diminution in the redness, an exudation of lymph, and removal of slough and débris. The remote effects are, briefly, healing. According to the author, ulcers that have resisted all ordinary forms of treatment for weeks or even months show signs of improvement within twenty-four hours of the first application of the treatment.

### Gastrospasm.

Formerly spasms—enterospasm, gastrospasm—were assigned an important position among the morbid conditions of the alimentary tract. The advances in surgery have demonstrated that the diagnosis of spasms was made to cover a number of conditions, in which some more definite organic lesion was involved, and in consequence the tendency has been to ignore it altogether. More recently, however, it has been pleaded that the discredit into which the old doctrine of spasmodic contraction of the intestine had fallen has been exaggerated, and according to Dr. Waldvogel, the same applies to gastrospasm. He contributes an article on the subject to the *Münchener Medizinische Wochenschrift.* The symptoms of gastrospasm are chiefly a feeling of weight immediately after a meal, accompanied by gaseous eructations. Objectively the spasmodic contraction of the stomach can be demonstrated by the injection of sodium bicarbonate and tartaric acid three or four hours after a meal, with the patient in a recumbent position. On percussion the distended stomach should normally reach the umbilicus or below it, but in the condition of gastrospasm the lower margin only reaches to within two or three fingers' breadth above the umbilicus. Of course, the normal dilatation of the stomach may be prevented by adhesions or by a neoplasm, but these eventualities can generally be determined by other indications. Gastrospasm occurs especially in individuals with an irritable weakness of the nervous system, accompanied by exaggerated reflexes, tremors of the hands and tongue, insomnia, rapid pulse, etc.; and it is also found in saturuism, tobacco-poisoning, arterio-sclerosis involving the abdominal organs, and in uræmia. For treatment of this condition the author prescribes atropine in pills twice or three times a day, and if this fails he orders in addition tinct. opii ♏.iij-v. three times a day. Neurasthenic symptoms should be treated by warm baths and proper regulation of diet, work, etc. In cases of arterio-sclerosis iodine may be administered in conjunction with the atropine. In tobacco-poisoning sudden and complete suppression of the tobacco may be followed by gastric atony and retention.

### Acute General Peritonitis.

In an interesting contribution to the *Journal of the American Medical Association*, Dr. Bevan, of Chicago, reviews the modern surgical treatment of acute general peritonitis and the various methods recommended by different authorities. Discussing the saline cathartic treatment of Tait, the rest and starvation method of Ochsner, the Murphy treatment with tubular drainage, continuous irrigation of the rectum, and the extreme Fowler position, he thinks that all these methods have their use in particular cases, but are capable of modification in certain points. He then proceeds to expound his own method, which he suggests is a happy combination of all the essentials. One of the first things to ascertain is whether the peritonitis is local or general, and this he does by means of a female catheter. If there is free pus widely disseminated it should be removed by irrigation. He condemns rubber tubes and prefers cigarette drains. The dressing should be wet with hot boric acid solution, and should be changed every six to twelve hours. The Fowler position, while of much value, has, he thinks, been used sometimes to the discomfort and disadvantage of the patient. All the possible benefit can be obtained by elevating the head of the bed 18 inches and placing a bolster, attached on both sides to the head of the bed, below the buttocks to prevent the patient from sliding. He is also of opinion that continuous irrigation of the bowel has been overdone. The same result can be obtained by interrupted injections of 6 to 16 ounces of normal saline every two to four hours. Repeated vomiting should be combated by gastric lavage and paralytic ileus by small repeated doses of castor oil.

### Determination of Sex.

At a recent meeting of the Académie des Sciences, Dr. R. Robinson discussed the relation of the internal secreting glands to pregnancy and especially to the determination of sex. It has already been shown that adrenalin is often efficacious in allaying the vomiting of pregnancy, and from this and other indications the theory has been formulated of a certain antagonism between the suprarenal and ovarion internal secretions. According to Dr. Robinson and Dr. J. Requault, who voiced similar views at the same meeting, vomiting and other troubles of pregnancy are in many cases due to an insufficiency of the suprarenals, and in their experience it is only in such cases in which there is other evidence of this insufficiency that administration of adrenalin is successful in suppressing the vomiting. They have found too that in such cases the woman gives birth to a female child. Dr. Robinson reports fifteen cases in which suprarenal insufficiency was evidenced by vomiting, disorders of nutrition, pigmentation, etc., and in which the birth of a female child resulted. Adopting the theory of crossed heredity, according to which the sex of the child is determined by the more vigorous of the parents in an inverse sense, Dr. Robinson suggests that the maternal parent is weakened by this suprarenal insufficiency, resulting accordingly in the birth of a female child. The hypothesis sounds attractive and fascinating. By a further knowledge of these internal secretions and their relative functions one might be able to determine the sex of the offspring by appropriate opotherapeutic measures.

### The Tonsils and Heart Disease.

That acute rheumatism is the most important factor in the causation of acute endocarditis and that tonsillitis not infrequently precedes or accompanies acute rheumatism, are two propositions which are universally accepted. It is not, therefore, strange to find Dr. Birfield arguing in the *Interstate Medical Journal* that the tonsils have a special ætiological relation to heart disease. It appears that Guerich was the first to propose amputation of the tonsil in all cases of acute articular rheumatism, and indeed at the first signs of the disease; this was in 1905 (*Gelenkrheumatismus*, Breslau). His method was that of guillotine tonsillotomy, subsequently abandoned in favour of the more thorough enucleation methods now taken into favour. This suggestion never commended itself to the profession, being somewhat of the nature of locking the stable after the horse has been stolen; and the mere fact that the patient is necessarily in a state of severe febrile infection contraindicates operative measures. Guerich, however, holds strongly that he can thus materially cut short the disease, and that the liability to cardiac complications is lessened; and a few other observers have corroborated this view. On the other hand, many physicians and pathologists believe that the tonsils are, as it were, the first line of defence against infection from without. Whether when chronically enlarged they are worth preserving for this purpose or should be sacrificed as having failed in their duty and become sources of danger, is a point on which opinion does not seem yet to have crystallised; their removal because of their mechanical interference with respiration or of their liability to recurrent inflammation is so frequently practised that their *rôle* as disposers to, or protectors against, heart disease is hardly taken into consideration.

### Spinal Bifida.

In the *Monthly Cyclopædia and Medical Bulletin* Dr. W. W. Babcock pleads for the early treatment of all babies with this abnormality by osteoplastic operation. He operates with the patient slung by the groins head downwards, and within the first few hours or days of life. A blanket is firmly fastened between the upright leg holders of an ordinary operating-table, so that from its upper edge the child may be suspended; the thighs are fastened down by a strap or bandage to prevent slipping. The object of this is to prevent too sudden a release of cerebro-spinal fluid when the sac is opened. Any abraded or ulcerated surfaces are painted with pure carbolic acid; then the operation field is disinfected, first with alcohol and then with iodine. 0.03 grain of novocaine dissolved in ½ c.c. of sterile 10 per cent. alcohol is then injected through a fine needle into the sac. This light solution will not reach any other part of the cerebral nervous system so long as the head down position is maintained. The sac is then resected if possible; but if there are too many nerve filaments incorporated with it, it is permitted to collapse into the spinal cavity, folded there, and then sutured across. Then the dura mater where it blends with the inner surface of the lamina is incised and stripped from the bony canal; it is then sutured together over the cord, thus restoring the dural canal. Then the laminæ are divided with bone forceps for the entire length of the defect, after which they, too, are sutured together in the mid line over the dura mater. The same is done with the erectorspinæ group of muscles, and finally the skin also is sutured across without any drainage. Two out of four babies thus treated have flourished; the other two survived the operation, but died later of hydrocephalus. Of the two survivors both are in fairly good condition. One has club foot, but is robust and bright mentally; the other has some anæsthesia, but is otherwise quite a healthy child. The shock is said to be less than one would expect.

### A New Sign of Scarlet Fever.

In February last a new help in the diagnosis of scarlet fever was described in the *Presse Medicale* by Pastia, of Bucharest. This sign has now been tested by Dr. Taubles, who contributes a paper about it to the *California State Journal of Medicine*. The sign consists of an intense continuous linear pigmentation of the skin folds across the anterior surface of the elbow; the colour varies from rose red to claret colour, and may be ecchymotic. The lines vary from one to four in number; between them the ordinary punctate eruption may be visible. This sign appears about the same time as the rest of the eruption, but it lasts much longer, and may even be seen in some cases when desquamation is nearly complete. Dr. Taubles and some of his colleagues have found this sign in every one of twenty-three cases of scarlet fever; it has been found the simple manœuvre of pressing the skin to cause momentary pallor assists in observation, as the red lines then stand out better against the whiter skin. He finds, however, that though in measles the sign is generally absent, still it is seen now and then, which detracts considerably from its diagnostic value. In many other diseases liable to be confused with scarlet fever he failed to elicit Pastia's sign; these include erythema from drugs, suppurative tonsillitis, acute catarrhal tonsillitis, and pharyngitis, acute pulmonary tuberculosis, acute appendicitis, and erysipelas. It must be added that the measles cases in which it has so far been seen are of the very severe, verging on the hæmorrhagic type, in which there is less opportunity for doubt as to the diagnosis than in milder cases. There seems to be a consensus that the sign is found in almost 100 per cent. of actual scarlet fever cases.

### The Organism of Scarlet Fever.

The investigations carried out by E. C. Schultze, of New York, for the past three years, with reference to the micro-organisms to be found in throat secretions in scarlet fever are reported in the *Medical Record*. He has obtained the secretions direct from the post-nasal space, and not from the tonsils, and in 459 out of 555 cultures obtained by this method has succeeded in isolating a large coccus which he considers the causal agent of scarlet fever. This organism was found in the blood serum of a child dying from the disease. It was not present in the desquamated epithelium of seven cases, but has been found in the pus from the eye and ear in scarlet-fever cases. It is present for from two to six weeks after the onset of the disease, but has been discovered as late as six months after. The author has not been able to find it in secretions obtained from healthy children's throats, though he has examined over a hundred. He has not been able to produce the disease by inoculation with the supposed causal organism, though five pigs so inoculated showed rash, fever, malaise, etc.

